# Localized Tacrolimus Delivery Repairs the Damaged Central Nervous System

**DOI:** 10.1016/j.ebiom.2017.11.026

**Published:** 2017-11-28

**Authors:** Stephanie Michelle Willerth

**Affiliations:** a Department of Mechanical Engineering, University of Victoria, Victoria, B.C., Canada; b Division of Medical Sciences, University of Victoria, Victoria, B.C., Canada; c Centre for Biomedical Research, University of Victoria, Victoria, B.C., Canada; d International Collaboration on Repair Discoveries (ICORD), Vancouver, B.C., Canada

**Keywords:** Controlled release, Neuroscience, Regeneration, Electrospinning, Nanofibers

The central nervous system (CNS) possesses a limited capacity to regenerate in comparison to other tissues with higher regenerative capacities, like the peripheral nervous system and skin. Thus, CNS injuries can be particularly devastating as the body does not repair these wounds in the same manner as these other tissues. Damage to the CNS comes in two waves post-injury. The initial trauma causes the primary injury, disrupting structures of the CNS and leading to cell death. A cascade of events follows this initial injury, causing additional damage and reducing the regenerative capacity of CNS tissue. These events include scarring of the wound induced by astrocytes and infiltration of the injury site by neutrophils and macrophages due to the immune response. Both of these phenomena contribute to the inhibitory environment present after injury. Reducing the effects of the inflammatory response increases the potential for regeneration after such injuries by improving the microenvironment. The Food and Drug Administration (FDA) approved drug tacrolimus, also known as FK506, acts to suppress the immune system. Accordingly, it is delivered either systematically or orally to prevent organ rejection after transplantation. Tacrolimus also promotes nerve regeneration through the aforementioned mechanism by mitigating the effects of inflammation and reactive glia, including astrocytes (Konofaos and Terzis, 2013). However, systematic administration of the levels of tacrolimus necessary for promoting regeneration in CNS results in damage to other organs in the body that take up the drug more readily (Kruh and Foster, 2012).

Controlled release of tacrolimus from biomaterial scaffolds addresses this issue by providing localized delivery of this agent to the injury site while limiting its exposure to other organs. However, previous biomaterial-based controlled release strategies have focused on tacrolimus delivery to other tissues, like skin, using hydrogels, (Gabriel et al., 2016) or by using microspheres to promote peripheral nerve regeneration *in vitro* (Tajdaran et al., 2015) – instead of attempting to regenerate damaged CNS. The study by van der Merwe et al. featured in this issue of EBioMedicine develops a novel electrospun scaffold for *in vivo* tacrolimus delivery (van der Merwe et al., 2017). Electrospinning serves as a commonly used method for producing defined nanofiber scaffolds with appropriate physical and chemical properties for tissue engineering applications (Villarreal-Gómez et al., 2016).

Here, they fabricate these fibrous scaffolds from the low cost, biocompatible elastomeric material poly(ester urethane)urea (PEUU) (Stankus et al., 2004) and tune the mechanical properties of this electrospun scaffold referred to as PEUU-Tac to replicate the features of the dura, the protective membrane that surrounds the CNS. They first loaded tacrolimus into these scaffolds and showed that these scaffolds deliver the majority (~ 85%) of the drug within 24 h as indicated by *in vitro* release studies. These scaffolds remained stable for 5 weeks before starting to degrade. They also showed that tacrolimus modulates the survival of retinal ganglion cells in a dose-dependent fashion *in vitro* and increases neurite extension in the same cell culture system. These electrospun sheets can function as an artificial dura for repairing the damaged CNS. In particular, this study focused on repairing the optic nerve after an ischemia injury model in rats. These scaffolds were wrapped around damaged optic nerves followed by suturing to ensure they remained in place. The animals were then monitored to determine the effect of these drug releasing scaffolds on nerve regeneration while simultaneously measuring the systemic release of tacrolimus. Controlled delivery of tacrolimus reduced the expression of glial fibrillary acidic protein (GFAP), suggesting a decrease in glial scar formation. It also increased GAP-43 expression, suggesting regeneration of the retinal ganglion was occurring *in vivo*. These scaffolds were also easily removed after 2 weeks as the majority of the drug was released in the initial 24 h. Thus, they can be easily replaced if necessary to extend the duration of release. Importantly, the authors showed that the levels of tacrolimus in the blood were not significantly elevated, demonstrating the benefits of using this controlled delivery strategy. Future work includes optimizing the release profile of tacrolimus from these scaffolds due to its narrow working range of concentrations, and assessing if these scaffolds can promote functional recovery in pre-clinical CNS injury models.

This study demonstrates a novel, clinically-relevant strategy for localized delivery of tacrolimus for repairing the damaged CNS. It achieves an important milestone towards adapting such a strategy for clinical applications. Surgeons could easily use these PEUU-Tac scaffolds as novel patches that mimic the dura and its properties to repair damaged optic nerve *in vivo*. These novel scaffold properties also suggest that this regeneration-promoting strategy could be implemented throughout the CNS, including for applications in repair of the damaged spinal cord or in the case of traumatic brain injury. Future studies will investigate how to properly balance the necessary delivery of tacrolimus to achieve regeneration while avoiding its negative side effects that occur due to differential uptake by other tissues of the body.

## Disclosure

The author declared no conflicts of interest.
